# Beyond traditional sleeping pills: safety, efficacy and multidisease potential of three approved orexin receptor antagonists

**DOI:** 10.3389/fpsyt.2026.1864080

**Published:** 2026-08-18

**Authors:** Zening Yang, Chenshuo Jia, Zhizhao Ma

**Affiliations:** 1 Department of Neurosurgery, Hebei General Hospital, Hebei Medical University, Shijiazhuang, China; 2 The First Hospital of Hebei Medical University, Shijiazhuang, China; 3 Department of Neurosurgery, The Second Hospital of Hebei Medical University, Shijiazhuang, China

**Keywords:** anti-insomnia drugs, efficacy, orexin receptor antagonists, safety, sleep disorder

## Abstract

This is a narrative review aiming to summarize the latest research progress of orexin receptor antagonists in insomnia treatment. To review the latest research progress on orexin receptor antagonists in the treatment of sleep disorders and explore their potential in terms of drug safety, efficacy, addiction risk, and the treatment of insomnia comorbid with psychiatric and neurological disorders. Orexin receptor antagonists bind to orexin receptor-1 (OX1R) and/or orexin receptor-2 (OX2R) receptors, block the orexin-mediated arousal signaling pathway, reduce the body’s arousal drive, and thereby improve sleep continuity. Orexin receptor antagonists regulate orexin secretion from the hypothalamus, improving sleep quality and maintaining a normal sleep structure. At present, there are still research gaps in multiple application directions. As a class of agents with unique mechanisms of action, orexin receptor antagonists have promising prospects in the treatment of insomnia. Currently, only three orexin receptor antagonists have been approved for clinical use. Research remains limited, and further exploration is needed in some application areas. As a new class of drugs with a unique mechanism, orexin receptor antagonists show great potential in the treatment of insomnia. Future studies should focus on optimizing their application strategies and addressing current research gaps to provide more comprehensive solutions for sleep disorder management.

## 1 Introduction

Insomnia is a common form of sleep disorder, characterized by difficulty in falling asleep, difficulty in maintaining sleep, or poor sleep quality, accompanied by obvious daytime symptoms such as fatigue, inattention, cognitive decline, irritability, anxiety, and low mood (1). Statistically, approximately 30% of the global population suffers from insomnia. Insomnia can occur alone or co-occur with mental, metabolic, or cardiovascular diseases, which undoubtedly highlights the importance of treating this complex condition (2).

Sleep disorders are a broad concept, encompassing many abnormal conditions that affect sleep quality, sleep duration, sleep rhythm, and physiological processes related to sleep. Professional classification systems such as the World Health Organization’s International Classification of Diseases and the American Psychiatric Association’s Diagnostic and Statistical Manual of Mental Disorders (DSM) have detailed classifications of sleep disorders. Common types of sleep disorders include sleep apnea syndrome, narcolepsy, restless legs syndrome, circadian rhythm sleep disorders, and insomnia disorders, etc. The core of the discussion in this paper will focus on this specific type of insomnia disorder (3).

Currently, the treatment strategies for insomnia mainly focus on two major areas: cognitive therapy and drug therapy. When Cognitive Behavioral Therapy for Insomnia (CBT-I) is unavailable, yields insufficient therapeutic effects, bears contraindications, or short−/medium−term pharmacological intervention is clinically required, dual orexin receptor antagonists (DORAs) can serve as evidence−based pharmacotherapeutic options. Clinical practice guidelines issued by the American Academy of Sleep Medicine (AASM) may be cited to clarify the position of drugs such as suvorexant within the overall diagnosis and treatment system for insomnia (4). Traditional benzodiazepines are often used as anti-insomnia drugs in clinical practice, but certain side effects and limitations have been exposed during long-term application. For example, in the treatment of chronic insomnia, factors such as safety, tolerance, variability of efficacy, and the risk of drug abuse limit the use of benzodiazepines (5–7). Research shows that patients who take benzodiazepines for a long time are prone to developing psychological and physical dependence, and some patients will experience severe withdrawal symptoms after stopping the drug (8). Therefore, for patients who have been taking benzodiazepines for a long time, in order to avoid the “withdrawal rebound” phenomenon, a slow-withdrawal method is required (9).

In contrast, DORAs have been proven to promote the sleep process in various organisms, including humans. DORAs provide the body with more precise and sufficient sleep by maintaining the normal sleep structure. It is worth noting that in the research on orexin receptor antagonists, neither drug dependence and addiction nor adverse reactions such as respiratory depression have been found (10). For specific vulnerable groups such as the elderly, DORAs may have more advantages in terms of safety compared with benzodiazepines.

This review systematically compares the three clinically approved DORAs, namely suvorexant, lemborexant and daridorexant, against conventional hypnotic agents. It conducts a comprehensive analysis covering their mechanisms of action, pharmacokinetic profiles, effects on physiological sleep architecture, clinical efficacy, overall safety profiles and abuse/dependence risks, as well as their therapeutic potential in insomnia patients comorbid with psychiatric and neurological disorders. This work intends to summarize the research progress of orexin receptor antagonists and offer novel evidence and references for the clinical management and new drug development of insomnia disorders.

## 2 Sleep disorders and orexin receptor antagonists

### 2.1 Literature search strategy

This narrative review was assessed in accordance with the SANRA (Scale for the Assessment of Narrative Review Articles) framework. After revision, the manuscript obtained a total score of 12 out of 12, fully meeting the methodological standards for high-quality narrative reviews. No systematic quantitative meta-analysis following PRISMA guidelines was performed, and the rationale for selecting a narrative synthesis design is elaborated in the response to reviewers.

### 2.2 Sleep structure and cycles

**Figure 1:**
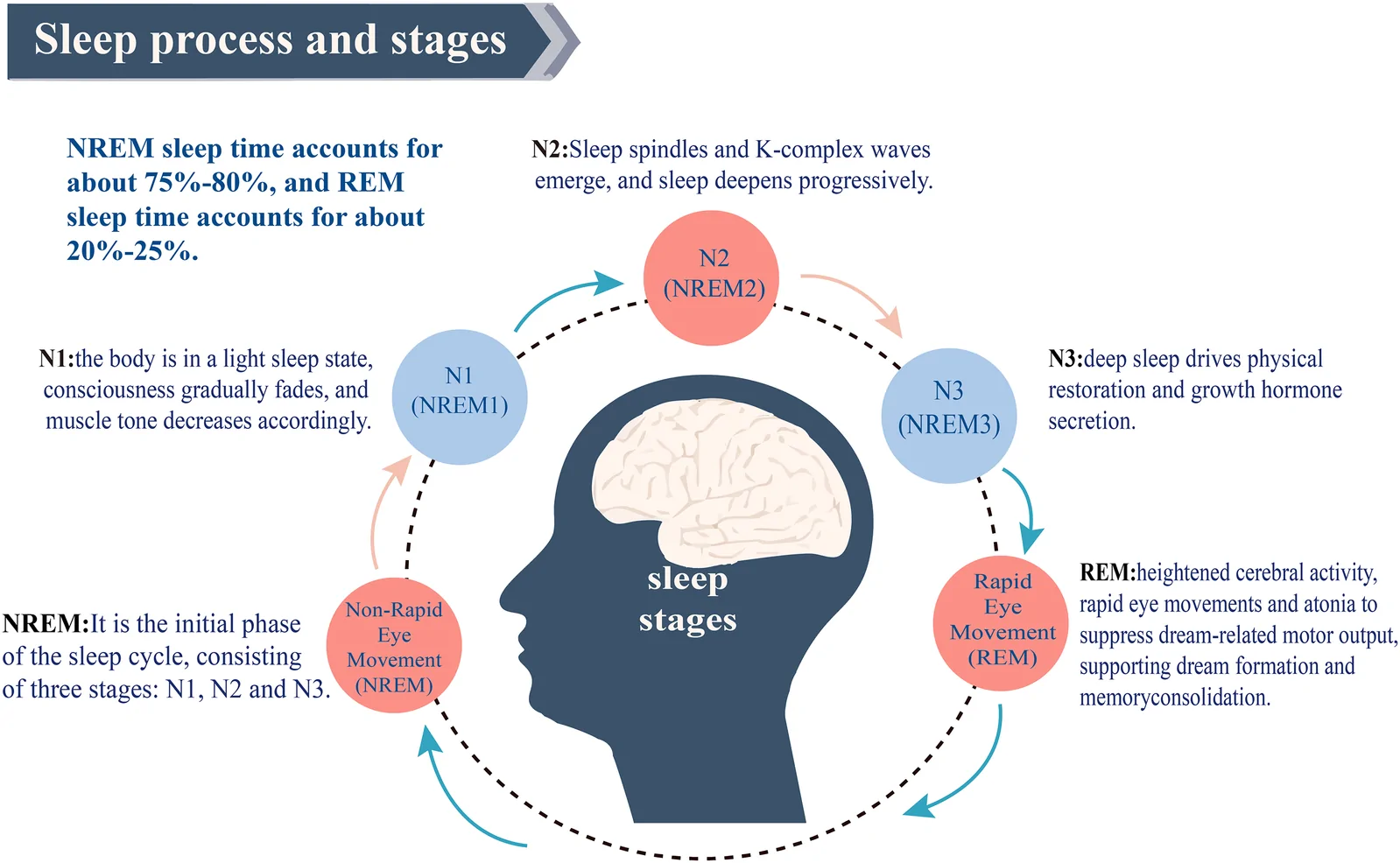
Sleep stages and sleep cycles in humans. A complete single sleep cycle lasts approximately 90–110 minutes, with alternating NREM sleep and REM sleep.

Human sleep cycles are categorized into non-rapid eye movement (NREM) and rapid eye movement (REM) sleep, with NREM further subdivided into N1, N2 and N3 slow-wave deep sleep (11–14). N1 represents light sleep initiation with diminished consciousness and muscle tone; N2 is marked by sleep spindles and K-complexes that signal sleep deepening; N3 deep sleep drives physical restoration and growth hormone secretion. REM sleep features heightened cerebral activity, rapid eye movements and atonia to suppress dream-related motor output, supporting dream formation and memory consolidation. A full physiological cycle spans 90–110 min and repeats overnight, with progressive deepening of NREM prior to each REM phase (see **Figure 1**). Disrupted sleep architecture, commonly induced by circadian disruption, screen overexposure and chronic stress, predisposes individuals to insomnia, narcolepsy and sleep-disordered breathing (15). Persistent sleep cycle dysfunction further elevates the risk of cardiometabolic and mood comorbidities (16). This orderly multi-stage sleep framework is tightly regulated by the hypothalamic orexin arousal system, making intact sleep architecture a critical therapeutic endpoint for insomnia pharmacotherapy. Unlike benzodiazepines and non-benzodiazepine GABA modulators that distort deep and REM sleep, DORAs normalize hyperarousal via orexin pathway inhibition while preserving physiological sleep staging. Clarifying stage-specific sleep physiology therefore lays a foundational framework for comparing how distinct hypnotic classes remodel sleep structure.

### 2.3 The relationship between orexin and the sleep-wake cycle

Insomnia is closely associated with an abnormal state of hyper-arousal, which is manifested as over-excitement and hyper-vigilance at the physiological, psychological, and cognitive levels. Orexin is a neuropeptide secreted by the hypothalamus, which plays a crucial role in regulating the sleep-wake cycle, orexin, energy balance, and the reward system, etc. (17). Research indicates that orexin can promote wakefulness and maintain a state of alertness in sleep regulation. When the orexin level in the human body is high, an individual is more likely to stay awake. During sleep, the orexin level decreases, which is closely related to its physiological function of promoting wakefulness and alertness. Therefore, orexin is regarded as an important substance for promoting wakefulness and alertness.

The orexin system plays a pivotal role in regulating the human body state. It can flexibly adjust the degree of arousal according to the different needs of individual behaviors. Relevant research points out that the orexin system is not only involved in the regulation of the wake-state but also plays a role in stress release (18). Specifically, DORAs can regulate the fear-related neural pathways and physiological responses by inhibiting orexin receptors, thus alleviating fear to some extent. Although there is currently no conclusive direct evidence to prove that the orexin system in insomniacs is in a state of hyper-activity, it is reasonable to speculate that the imbalance of the orexin system may be related to the hyper-arousal state associated with insomnia.

It is noteworthy that a recent study on mice has made an interesting discovery: as the mice age, the number of orexin neurons in their bodies gradually decreases, while the remaining neurons show a state of hyper-excitation. Eventually, this leads to a general decrease in the awakening threshold during sleep and sleep fragmentation in mice (19). Although this experiment is not a traditional insomnia model, this result still helps to reveal the relationship between some sleep problems in the elderly and the orexin system.

### 2.4 The development of orexin receptor antagonists

In 1998, de Lecea et al. and Sakurai discovered (20, 21) orexin, which was named for its ability to enhance food intake. Subsequent studies have shown that it has diverse physiological functions. In recent years, the number of research papers on the distribution, molecular mechanisms, physiological functions, disease associations, and drug development potential of orexin and its receptors has gradually increased. Orexin signaling is crucial for regulating wakefulness and alertness. Abnormal function is associated with narcolepsy. Due to its high selectivity, the signaling pathway has become an attractive target for treating sleep disorders such as insomnia (22).

**Figure 2:**
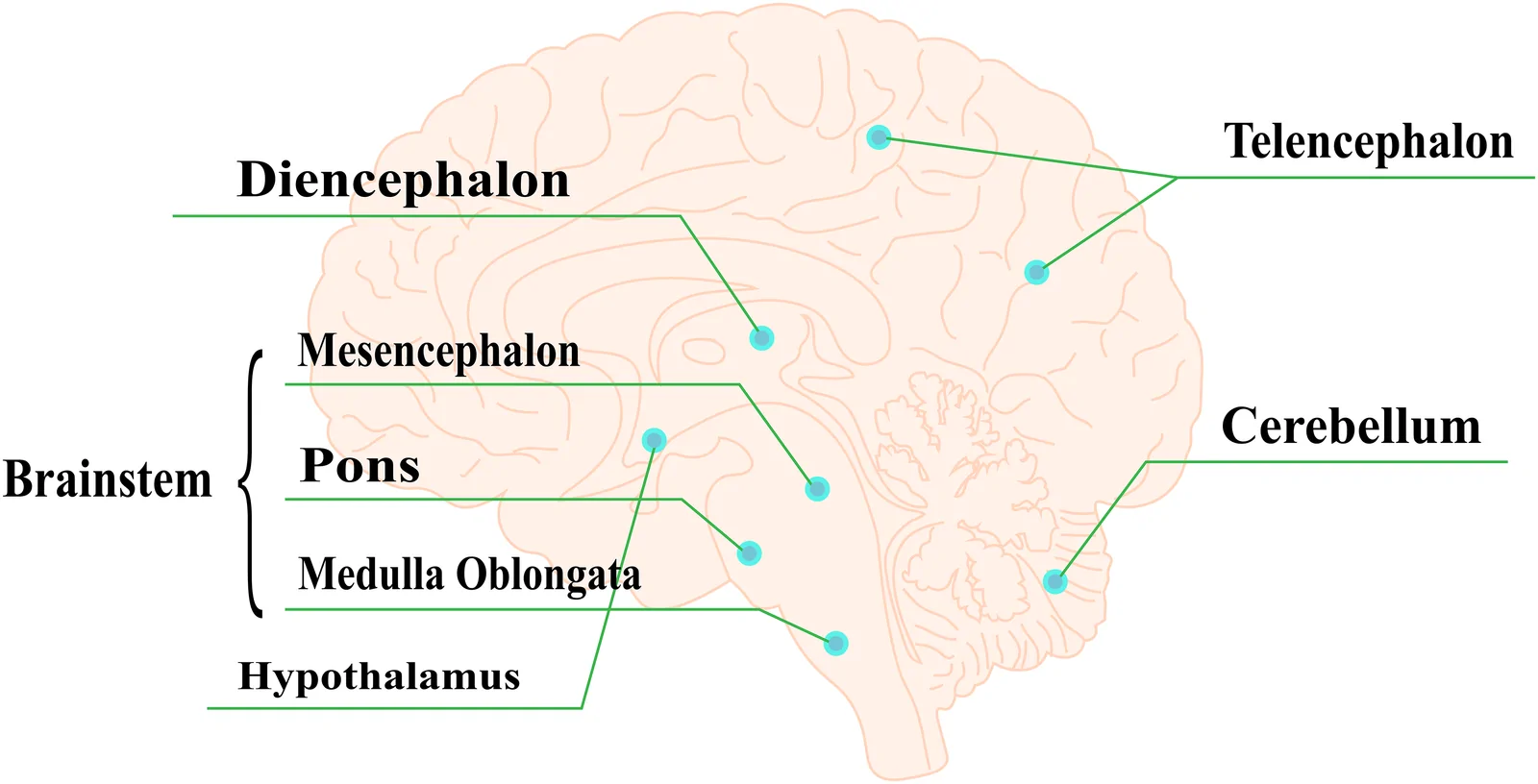
Orexin-A is a neuropeptide secreted by the hypothalamus. It is synthesized in the lateral hypothalamic area and widely distributed throughout the central nervous system. It exerts diverse physiological functions by binding to G protein-coupled receptors OX1R and OX2R.

At the same time, research on the application of orexin in the treatment of mental diseases has increased, covering sleep disorders, depression, anxiety, and drug addiction, etc. (23). For example, in sleep disorders, OX2R agonists can improve cataplexy-like attacks in narcoleptic mice. OX1R and OX2R have different roles in sleep regulation. OX2R is crucial for regulating wakefulness and NREM sleep, while REM sleep is jointly regulated by both. In terms of mood disorders, orexin receptor antagonists are related to the treatment of depression and anxiety. Acute restraint stress activates orexin neurons in the lateral hypothalamus, increasing the level of orexin-A in the midbrain ventral tegmental area region (see **Figure 2**). Moreover, OX1R gene variations are associated with the development and severity of major depressive disorder.

Kumar et al. (24) emphasized the new role of orexin antagonists in the treatment of insomnia, paying attention to their physiological characteristics and development as a new strategy in sleep medicine. Han et al. also explored the role of the orexin system in mental diseases, pointing out that research on the treatment of related diseases with its receptor antagonists is gradually increasing.

**Figure 3:**
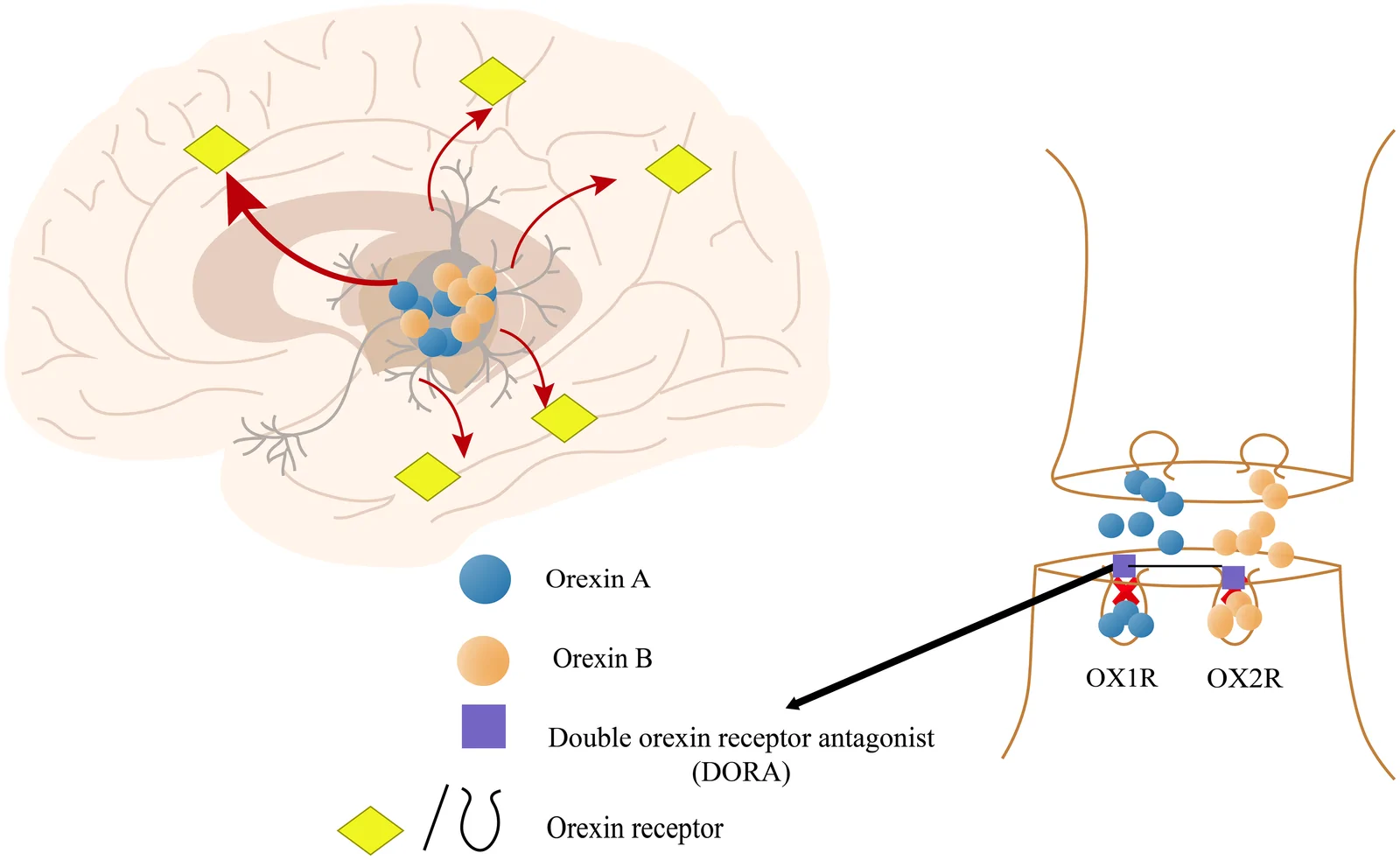
Physiological mechanism of orexin mechanism of orexin.

The orexin family consists of two neuropeptides, orexin-A and orexin-B, which bind to OX1R and OX2R respectively. According to the binding affinity, antagonists are divided into selective orexin receptor 1 antagonists, selective orexin receptor 2 antagonists, and DORAs (25). OX2R is crucial in sleep regulation and also cooperates with OX1R to regulate REM sleep (25) (see **Figure 3**).

Insomnia is closely associated with hyper-arousal. Physiologically, hyper-arousal causes the nervous system to remain continuously excited, making it difficult for the body and brain to transition from an active state to sleep (23). For example, sympathetic nerve excitation increases heart rate and blood pressure, which is not conducive to falling asleep. In terms of neurochemistry, excessive secretion of wake-promoting neurotransmitters (such as norepinephrine and dopamine) or an imbalance of sleep-regulating neurotransmitters (such as serotonin and gamma-aminobutyric acid (GABA)) can trigger hyper-arousal and lead to insomnia (26). Psychologically, long-term anxiety, stress, etc. cause hyper-arousal and disrupt sleep (27). In terms of sleep structure, hyper-arousal disrupts the sleep cycle, reduces deep sleep and REM sleep, and exacerbates insomnia. Hyper-arousal is an important factor in the occurrence and persistence of insomnia, forming a vicious cycle with insomnia (28).

Three orexin receptor antagonists, suvorexant, lemborexant, and daridorexant, have been approved for the treatment of insomnia (29–31). Based on a comprehensive analysis of preclinical research and data from marketed drugs, this paper finds that excessive activation of the arousal system may be one of the key predisposing factors underlying the pathogenesis of insomnia. Traditional drug intervention methods that enhance sleep signals [such as modulating the gamma-aminobutyric acid type A receptor (GABAA)] may not be the best in terms of physiological mechanisms. In contrast, emerging DORAs drugs can reversibly weaken the over-arousal signal through a unique mechanism, providing a new approach to the treatment of insomnia.

**Figure 4:**
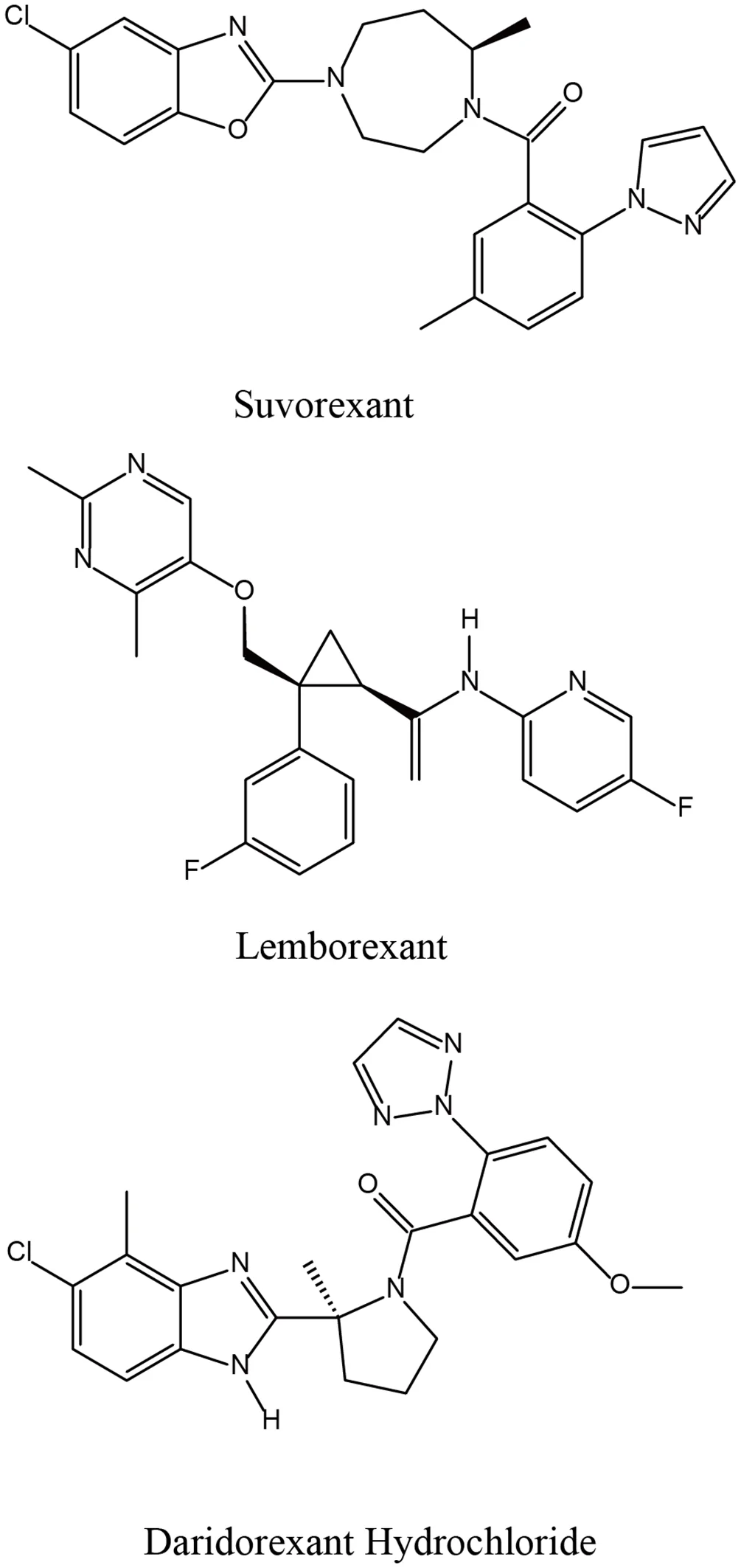
Molecular structural formula of three dual orexin receptor antagonists.

Over the past two decades, numerous pharmaceutical companies worldwide have conducted in-depth research on orexin receptors and developed a large number of related compounds. Among them, as for selective orexin receptor 1 antagonists, current research results show that they have not yet demonstrated significant effects in improving insomnia symptoms (32). Although some selective orexin receptor 2 antagonists have shown strong hypnotic effects in animal experiments, these results still need to be further verified through clinical research to confirm their actual effectiveness (33). In this article, we will focus on three novel orexin receptor antagonists for insomnia treatment, namely suvorexant, lemborexant and daridorexant (see **Figure 4**).

### 2.5 The effects of orexin receptor antagonists and traditional insomnia drugs on sleep stages

In the field of insomnia treatment, benzodiazepines are regarded as traditional and highly influential “star drugs” and are widely used due to their specific pharmacological mechanisms and remarkable clinical efficacy. Precisely because of their important position in this field, many newly launched drugs, including orexin receptor antagonists, generally take them as a key reference for comparison. Through this comparison, various characteristics of orexin receptor antagonists, such as their effects on different sleep stages, are evaluated, and then their advantages and disadvantages compared with benzodiazepines can be clarified.

Benzodiazepines such as diazepam can shorten the sleep latency and increase the total sleep time. However, they may reduce the proportion of deep sleep and have a certain inhibitory effect on REM sleep. Long-term use may also lead to dependence and residual effects (34). Non-benzodiazepines such as zolpidem mainly act to shorten the sleep-onset time and increase sleep efficiency. They have a relatively small impact on NREM sleep and a milder inhibitory effect on REM sleep compared with benzodiazepines (35).

Non-benzodiazepine drugs are a class of medications used to treat insomnia and anxiety symptoms. They differ from traditional benzodiazepines in chemical structure, thus having different mechanisms of action and side-effect profiles. These drugs mainly exert their sedative-hypnotic effects by enhancing the activity of GABA receptors. Similar to benzodiazepines, but with more specific actions, they usually target specific subtypes of GABA receptors. This mechanism helps reduce excessive brain excitation, thus achieving the effects of promoting sleep or alleviating anxiety (36). Among them, non-benzodiazepine drugs have achieved good therapeutic effects in acute insomnia. However, long-term use of these drugs has many side effects, including cognitive impairment, tolerance, rebound insomnia after discontinuation, traffic accidents-falls, abuse, and dependence liability, etc. In addition, long-term use often leads to abnormalities in sleep structure (37).

Most non-benzodiazepine drugs have fewer side-effects because they are selective for GABAA receptors containing the α1 subunit (38). Taking zolpidem, zopiclone and zaleplon as examples, these drugs not only have hypnotic effects similar to those of benzodiazepine drugs but also have good safety. Compared with benzodiazepine drugs, non-benzodiazepine drugs have less adverse effects on normal sleep structure, psychomotor and memory functions, and rarely have rebound insomnia and withdrawal symptoms after drug withdrawal (39). In the long-term treatment of insomnia, zolpidem and zopiclone have good tolerance and are relatively suitable choices, but their long-term efficacy and safety still need to be further explored. In addition (40), considering that the degree of respiratory depression caused by non-benzodiazepine drugs is very slight, for patients with respiratory diseases, their safety may be better than that of benzodiazepine drugs (40).

Different from traditional drugs, current research shows that orexin receptor antagonists (ORAs) have high selectivity. They mainly target orexin receptors and have relatively low direct interference with other neurotransmitter systems. Theoretically, ORAs are expected to show more precise therapeutic effects. In terms of side effects, they may mainly focus on areas related to sleep regulation, such as possible mild daytime sleepiness, dizziness and other symptoms. However, compared with some traditional hypnotic drugs, the impact of ORAs on cognitive and respiratory functions is usually relatively milder.

In terms of sleep regulation, the way ORAs act on sleep stages is not to directly inhibit neuron activity, but to act in a way that is more conducive to improving insomnia. Specifically, this type of drug can significantly increase the duration and quality of NREM sleep, especially the N3 stage (deep sleep), which is of great significance for physical recovery and function restoration (41). At the same time, ORAs can reduce the number of nocturnal awakenings and shorten the awakening duration, making sleep more coherent and stable, and improving the overall sleep continuity. In addition, the impact of ORAs on REM sleep is relatively limited, generally not significantly changing its proportion and cycle, which helps to maintain the body’s natural sleep rhythm (42).

## 3 The clinical efficacy of orexin receptor antagonists

### 3.1 Evaluation criteria for insomnia

Insomnia is a chronic clinical disorder, and its accurate diagnosis requires a combination of subjective judgment and objective assessment. Reliable sleep quality assessment and standardized indicators are crucial for determining the efficacy of insomnia medications. Commonly used assessment indicators include wake time after sleep onset (WASO), latency to persistent sleep (LPS), total sleep time (TST), subjective total sleep time (sTST) and sleep efficiency (SE). These indicators are typically measured and recorded through polysomnography (PSG) or sleep diaries (43).

Polysomnography is a comprehensive sleep monitoring technique that can simultaneously record multiple physiological parameters such as brain waves, eye movements, and muscle activities, allowing for precise assessment of sleep quality and structure. Sleep diaries, on the other hand, are self-recorded by patients, documenting sleep onset time, wake-up time, and the number of nocturnal awakenings, among other sleep conditions. Although relatively simple, they still provide important information. In the research and treatment of sleep disorders, these assessment methods and indicators play a significant role.

### 3.2 Validity

For insomnia treatment drugs, their absorption and metabolism characteristics play a crucial role in the therapeutic process. The drugs need to take effect quickly and appropriately, and be eliminated in a timely manner, so as to maintain effective sleep for a long time at night without leaving residual drug effects in the morning (44, 45). For this reason, orexin receptor antagonists have attracted increasing attention due to their rapid and reasonable onset and elimination characteristics. After all, when patients receive sleep medication, they expect their daytime activities, such as traffic travel, business activities, and social interactions, to remain unaffected. In the field of insomnia treatment, in addition to addressing the issues of sleep initiation and maintenance, it is also necessary to pay attention to the third key aspect caused by insomnia, namely daytime functional impairment, which includes fatigue, drowsiness, comorbid mood disorders, and cognitive impairment (46, 47). If an insomnia treatment drug can significantly improve sleep conditions while having minimal impact on daytime functions, it is undoubtedly of great value. Because it not only effectively solves the core problem of insomnia but also ensures that patients can carry out their daily work and life normally (48).

**Figure 5:**
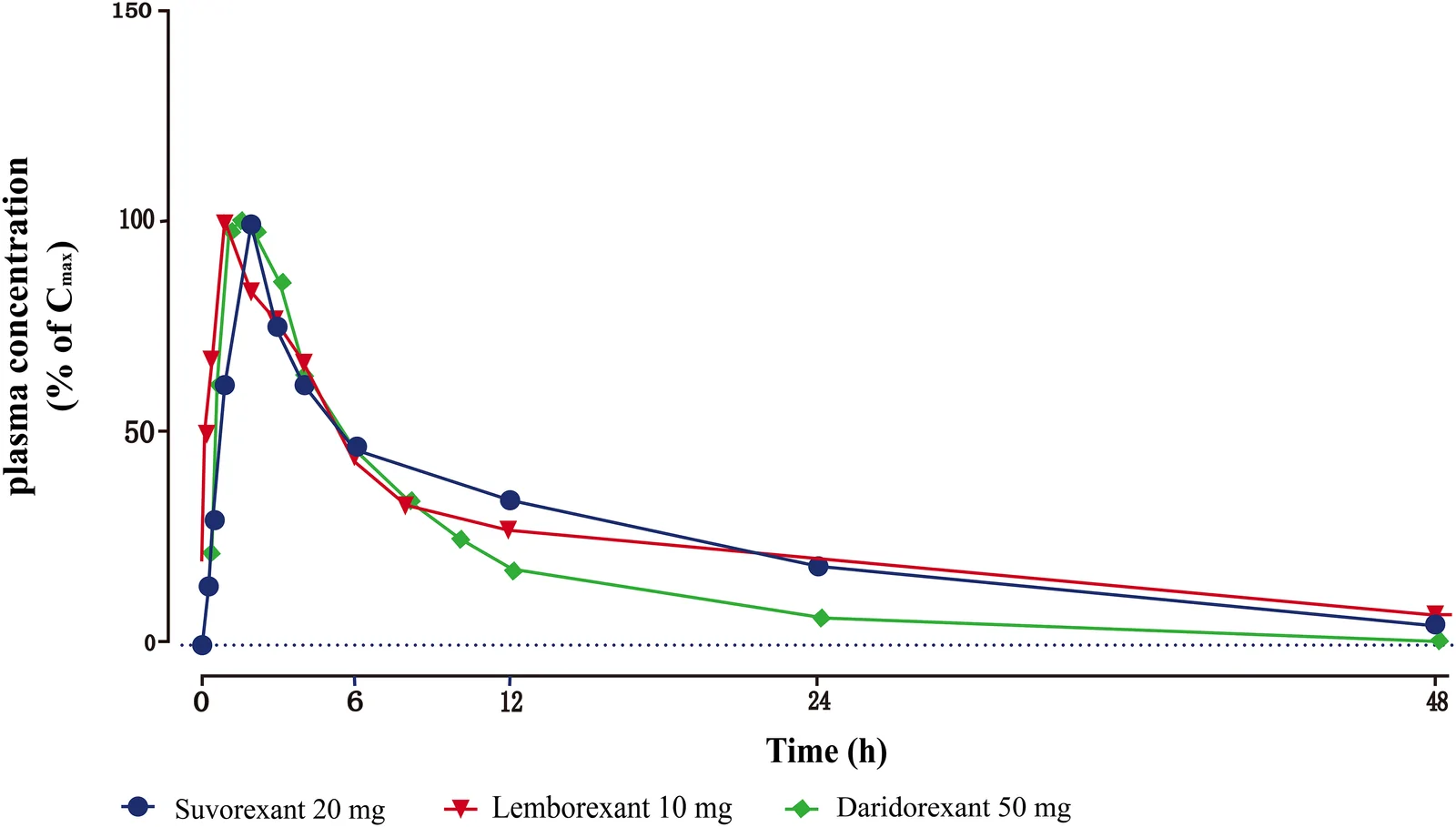
Maximum plasma Cmax-normalized plasma concentration-time profiles of suvorexant, lemborexant, and daridorexant (%). The data on the basis of which these profiles were constructed are taken from Food and Drug Administration (FDA) for suvorexant, Landry et al. (49) for lemborexant, Muehlan et al. (50) for daridorexant.

**Figure 5** shows some differences in pharmacokinetics (PK). The terminal elimination half-lives (t½) of daridorexant and suvorexant are 8 hours and 12 hours, respectively, while the reported effective t½ of lemborexant is 17 hours (terminal t½ is 55 hours), resulting in a plasma accumulation ratio of 2-3 (31, 51). Theoretically, prolonging drug elimination or increasing accumulation would lead to an increase in plasma levels, which might bring the risk of daytime sleepiness. However, there has been no report of an increased incidence of relevant hangover symptoms related to the DORAs category (52).

Besides the research on onset and elimination, long-term safety and efficacy are also particularly important and have been available for patients since 2014. It’s worth mentioning that in two Phase III trials conducted among more than 2,000 patients (with a treatment duration of 3 months), especially the previously unapproved 40/30 mg dose (for non-elderly/elderly patients), fewer patients in this experiment were randomly assigned to 20/15 mg (53). However, due to the lack of more high-dose experimental samples, the low-dose finally obtained FDA approval for marketing first.

In a study on the long-term efficacy, safety and tolerability of suvorexant, patients were followed up for one year. Compared with the placebo group, patients taking suvorexant had earlier sleep onset and longer sTST. A similar crossover study was conducted on young healthy men who underwent polysomnography and took 10, 50, and 100 mg of suvorexant on the second night. Similar to the previous study, LPS, sTST, and WASO all improved. The results showed that residual sleepiness on the second day was only significant at the 100 mg dose (54). Another experiment randomly assigned 249 patients to one of four different doses (10, 20, 40, and 80 mg) and had them take the medication for four weeks. The results showed that sTST, LPS, and WASO after sleep onset improved in a dose-dependent manner.

Previous studies have demonstrated the effectiveness of lemborexant. Objective evaluations of sleep were conducted at the 4th and 12th weeks after treatment. The data showed significant increases in sTST and SE at both the 4th and 12th weeks, indicating that lemborexant can effectively improve sleep duration and quality. Moreover, it has a long-term positive impact on the sleep quality of middle-aged and elderly patients with insomnia, not only subjectively but also objectively in terms of sleep parameters. It also has good safety and tolerability. This 40-week long-term extension trial confirmed that daridorexant had minimal next-day residual sedation. No withdrawal syndrome or rebound insomnia was detected after abrupt discontinuation. Improvements in sleep and daytime function remained stable throughout nearly one year of continuous treatment (55). Although a small number of subjects experienced drowsiness, this was the most common adverse event, and all adverse events were mild to moderate and transient, with no serious consequences (56).

In a secondary analysis of a randomized placebo-controlled trial of daridorexant, the efficacy and safety in insomnia patients of different age groups were deeply explored. Both 50 mg and 25 mg of daridorexant could significantly improve sleep after onset in both elderly and young insomnia patients, and the effect of the 50-mg dose was better than that of the 25-mg dose. In addition, 50 mg of daridorexant could also significantly improve the daytime functions of elderly and young insomnia patients, including sleepiness, alertness, cognition, and mood (57), etc. Another study found that in a retrospective observational study on the treatment of chronic insomnia, in daily clinical practice, the effectiveness of treating adult Chronic Insomnia Disorder (CID) showed that daridorexant was highly effective in CID treatment. It showed positive effects whether it was used for initial treatment, treatment after switching medications, or adjuvant treatment, and it had good tolerability, providing new evidence for clinical treatment (58).

In clinical trials, DORAs has been shown to be effective in improving sleep and maintaining sleep (44, 59). In the suvorexant and daridorexant validation studies, these benefits were replicated in similar independent studies over a 3-month period, further confirming the stable and effective role of DORAs in improving and maintaining sleep. .

### 3.3 Safety

Traditional hypnotic drugs have shown their drawbacks in terms of tolerance, dependence, complex drug interactions, and a relatively large number of adverse reactions. In contrast, DORAs show distinct differences from benzodiazepines in these aspects. The former (traditional hypnotic drugs) have been used in the medical field for many years, and as long as they are used rationally, their safety is relatively high. Generally speaking, when taking an appropriate dose, the adverse reactions of the drug can be controlled. The latter DORAs, as a relatively new type of drug, although current research shows promising prospects, more long-term clinical trials are still needed to further verify its long-term efficacy and safety. For example, it is currently uncertain whether some rare and delayed-onset adverse reactions will occur in the future. Comparing the safety of the two types of drugs and understanding their safety profiles, whether they are traditional or new drugs, helps to gain a more comprehensive understanding of their safety. We can see the differences from the comparison in **Table 1**.

**Table 1:** Pharmacokinetics, clinical efficacy, long-term safety and residual daytime sedation of three dual orexin receptor antagonists.

Item	Suvorexant	Lemborexant	Daridorexant
Terminal elimination half-life	12 h	Terminal t½= 55h; effective t½ = 17h, with a plasma accumulation ratio of 2–3	8 h
Theoretical risk of next-day somnolence	Moderate	Elevated (marked drug accumulation)	Low
Next-day hangover residual sedation	Significant drowsiness only observed at the high dose of 100 mg;	No hangover adverse events identified in clinical trials	Negligible next-day residual sedation
Improvement in sleep onset latency (LPS)	Dose-dependent shortening	Sustained objective improvements at Week 4 and Week 12	Significant improvements in sleep initiation and maintenance in both young and elderly patients
Total sleep time (sTST) and wake after sleep onset (WASO)	Progressive improvement with increasing doses	Long-term stable improvements in both subjective and objective sleep parameters in middle-aged and elderly patients	Sustained therapeutic benefits during nearly one year of continuous treatment
Stability of long-term administration (≥ 3 months)	Sustained sleep benefits during 1-year follow-up	Stable efficacy within 12 weeks with favorable tolerability	Consistent efficacy throughout the 40-week extension trial
Reactions after abrupt discontinuation	Withdrawal symptoms and rebound insomnia not systematically evaluated	No obvious rebound insomnia	No withdrawal syndrome or rebound insomnia
Improvement of daytime function in elderly populations	Daytime impairment only observed at ultra-high doses	Limited data	50 mg significantly relieves somnolence and improves alertness, cognition and mood
Adverse event profile	Mild and transient drowsiness; no serious adverse events	Mild adverse reactions and satisfactory tolerability	Somnolence was the most common event; all reactions were mild-to-moderate and transient
FDA-approved dose regimen	Only low doses (20 mg/15 mg) were approved; high doses (40 mg/30 mg) were rejected due to insufficient	Standard therapeutic doses	Two effective doses: 25 mg and 50 mg

#### 3.3.1 Addictiveness

As can be seen from **Table 2** for the DORAs that have been approved and launched on the market, there are many similarities in terms of safety. Research on the safety of long-term use of lemborexant in treating insomnia patients shows that patients who have undergone lemborexant treatment for 12 months or 6 months do not experience obvious rebound insomnia symptoms after drug withdrawal. The sleep outcomes within two weeks after drug withdrawal are similar to those at the 12th month of treatment, indicating that the positive effects of lemborexant can be maintained after drug withdrawal. Evaluated by the Tyrer Benzodiazepine Withdrawal Symptoms Questionnaire (T-BWSQ), almost no patients showed withdrawal symptoms, indicating that there is no obvious withdrawal effect after drug withdrawal (60). At the same time, the beneficial effects of DORAs in terms of improving the onset and maintenance of sleep have been verified. While this new type of prescription drug improves sleep quality, its side effects are significantly lower than those of traditional drugs.

**Table 2:** Comparison table of orexin receptor antagonists with benzodiazepines and non-benzodiazepines.

Drug categories	Orexin receptor antagonists			Benzodiazepines	Non-benzodiazepines
Names	Suvorexant	Lemborexant	Daridorexant	Diazepam	Zopiclone
Approved Countries	USA, Japan, etc.	USA, etc.	USA, Europe	Global	Global
Indications	Insomnia	Insomnia	Insomnia DSM	Anxiety, Insomnia	Insomnia
Mechanism of Action	Dual Orexin Receptor Antagonist	Dual Orexin Receptor Antagonist	Dual Orexin Receptor Antagonist	GABAA Receptor Enhancer	GABAA Receptor Subtype-Selective Enhancer
Safety	Common Adverse Effects. Drowsiness, headache Common adverse effects include drowsiness and headache	Drowsiness, urinary tract infection. Adverse effects include drowsiness and urinary tract infection	No significant dependency, low abuse potential, common adverse effects include headache, drowsiness, and dizziness	Side effects include drowsiness and memory impairment	Bitterness, drowsiness Side effects include bitterness and drowsiness
Efficacy	Improves sleep time and quality	Increases TST and improves sleep architecture	Significantly improves TST and WASO, limited effect on sleep latency	Strong, but prone to addiction	Improves sleep time and quality
Common Adverse Effects	Drowsiness, headache	Drowsiness, urinary tract infection	Headache, drowsiness, dizziness	Drowsiness, ataxia	Bitterness, drowsiness
Incidence	~10%	~12%	Unknown	High	~15%
Dependency/Addiction	Low	Low	No significant dependency or addiction risk	High	Moderate
T-max (h)	2	1-3	1-2	1-2	1-2
(Half-life (h))	12	17-19	8	~2	~6
WASO (min)	~30	~2 0	22-78	~40	~30
TST (min)	~60	~65	Increased by approximately 50–60 minutes	~50	~50
Sleep Latency (min)	~15	~10	No significant statistical difference	~20	~15
Other Parameters	Dose-dependent effects	More suitable for elderly patients	Improves daytime function, no significant residual effects	Long-term use prone to tolerance and dependency	Relatively safe for short-term use

As for the analysis of addictiveness in the table, the human body’s tolerance to the hypnotic effect of benzodiazepines (loss of drug efficacy) can develop within a few days. Similarly, physiological dependence on benzodiazepines develops after 3 to 6 weeks of treatment doses, meaning that sudden cessation will lead to rebound (worsening of original symptoms) or withdrawal. Under normal circumstances, the use of benzodiazepines should be short-term (61, 62). Guidelines in various countries recommend limiting the time for using them to treat anxiety or insomnia in adults to 2 to 4 weeks. This recommendation is based on data showing that these drugs begin to lose their efficacy for insomnia after 4 weeks, while the risks of side effects and addictiveness still remain (63, 64).

Benzodiazepines commonly used in clinical practice for the treatment of insomnia include alprazolam, estazolam, lorazepam, clonazepam, etc. These drugs are mainly applicable to adult patients with difficulty in falling asleep and sleep maintenance disorders, especially those with insomnia accompanied by anxiety disorders. Due to their properties of anti-anxiety, hypnotic, muscle-relaxing, and anti-convulsant effects, they are widely used. Doctors often use them to rapidly and short-term relieve insomnia, and the curative effect is remarkable. Most doctors may overlook their long-term risks due to the short-term benefits of benzodiazepines, resulting in these drugs still being widely used. In addition, clinical doctors may inadvertently underestimate these risks (65).

According to current research, a direction completely different from benzodiazepines has been discovered (66). The role of orexin in promoting reward and addiction is an emerging research field. Drug addiction is often associated with an increase in orexin system activity, suggesting that orexin receptor antagonists may be promising drugs for treating substance use disorders (67). Different from the gradually increasing addictiveness and dependence of benzodiazepine drugs, in the research on the addictiveness of DORAs, most studies mainly focus on the potential of orexin receptor antagonists in treating drug addiction, rather than whether the orexin receptors themselves can cause addiction. For example, the research includes the mechanism of action of the orexin system in the process of drug addiction, and how to use orexin receptor antagonists to intervene in addictive behaviors, such as exploring the effects of orexin receptor antagonists on drug intake and seeking behaviors when it comes to drug addictions like cocaine, alcohol, opioids, and nicotine. One study shows that orexin receptor antagonists can effectively reduce addiction-related behaviors in various animal models. For example, knocking down orexin expression or using orexin receptor antagonists can reduce the intake or seeking of cocaine, alcohol, opioids, and nicotine. This implies that regulating the activity of orexin receptors may be helpful in treating addiction, but it does not directly indicate that the orexin receptors themselves will not cause addiction (67). Therefore, treatment targeting the orexin system may become another promising research direction for reducing drug intake, decreasing the susceptibility to relapse, and restoring “normal” physiological functions, including eating and sleeping.

#### 3.3.2 Vulnerable groups and medication safety

When benzodiazepine drugs are used in vulnerable and special populations, there are limitations and potential risks. West et al. conducted a study on the compliance of clonazepam in the pain-management population, emphasizing the challenges of measuring low-concentration drugs (68). The use of these drugs in vulnerable populations may have limitations and risks, which should be further studied and considered in future clinical practice (69).

Both SUNRISE 1 (NCT02783729) and SUNRISE 2 (NCT02952820) have demonstrated that lemborexant has good tolerability. At the same time, we noticed that researchers from Eisai examined the impact of renal and liver impairment on the pharmacokinetics of lemborexant. For subjects with renal impairment, even in those with severe renal impairment, the pharmacokinetics of lemborexant was not significantly altered compared with healthy subjects with normal renal function (70). However, with a clearance rate decrease of approximately 20-35%, the exposure of lemborexant in subjects with liver impairment was significantly increased compared with healthy subjects (71). Therefore, for insomnia patients with liver function impairment, the selection of lemborexant dosage should be cautious.

Two recently published Phase 3 trials (NCT03545191 and NCT03575104 (44)) were not included in our analysis. The overall incidence of adverse events between any two treatment groups in the two trials was very close (in Trial 1, both the daridorexant 50-mg and 25-mg groups had an incidence of 38%, and the placebo group had 34%; in Trial 2, the incidence was 39% in the daridorexant 25-mg group, 38% in the daridorexant 10-mg group, and 33% in the placebo group), indicating that daridorexant has good tolerability. At the same time, we also found that daridorexant can be safely co-administered with other drugs, such as the antidepressant (72) and anti-anxiety drug citalopram, the cholesterol-lowering drug rosuvastatin (73), and the gastric pH regulator famotidine (74).

#### 3.3.3 Respiratory depression

Patients with impaired respiratory function should use benzodiazepines with caution because these drugs, acting on the central nervous system, may be associated with respiratory depression (52). In contrast, a study on the safety of DORAs was conducted in patients with Chronic Obstructive Pulmonary Disease (COPD) and Obstructive Sleep Apnea (OSA), who have a high incidence of insomnia symptoms. This study evaluated nocturnal respiratory function, including the apnea-hypopnea index and peripheral oxygen saturation (75). The results showed that DORAs had little effect on these indicators, indicating that the use of DORAs in patients with mild to moderate OSA or moderate COPD does not lead to adverse reactions related to respiratory depression. Although this study has some limitations, such as a small sample size and a short treatment duration (4–8 days), the preliminary results suggest that DORAs do not impair nocturnal respiratory function.

#### 3.3.4 Adverse reactions

Benzodiazepines also have serious adverse reactions, such as increased risk of falls, fractures, misuse, and dependence (61). These risks increase with the age of the patients and the duration of use. The risk of severe falls in people who have used benzodiazepines for a long time is 3% (62). Therefore, the American Geriatrics Society Beers Criteria recommends that the elderly should avoid using benzodiazepines (76), because adverse events are closely related to the frequency and dosage of these drugs. In sharp contrast, DORAs also show friendliness in the elderly population (57). The overall safety and tolerability of DORAs in elderly patients are basically the same as those in young patients, and there is no need to adjust the dosage according to age (77).

### 3.4 Role of orexin receptor antagonists in diseases associated with other sleep disorders

#### 3.4.1 Alcohol withdrawal

The orexin system also plays a role in the emotional dysregulation that occurs during alcohol withdrawal and alcohol-seeking behavior. In fact, the symptoms of acute alcohol withdrawal syndrome usually include sleep disorders. A recent systematic review has also linked these symptoms to orexin levels and neuroadaptive changes in the nucleus accumbens and prefrontal cortex (78). Preclinical and preliminary correlational studies have proposed the potential application of dual orexin receptor antagonists in managing insomnia symptoms during alcohol withdrawal. Current relevant conclusions are derived from neuroadaptive mechanistic hypotheses and small retrospective analyses, and high-quality randomized controlled trials are still required to verify its clinical efficacy in this population (79, 80).

#### 3.4.2 Anxiety disorders comorbid with insomnia

Anxiety-related disorders are the most common mental health disorders globally, with a global prevalence of approximately 25% (81). Anxiety disorders are accompanied by impairment of daily functioning, including Generalized Anxiety Disorder (GAD), panic disorder, etc. Insomnia is an important factor in anxiety attacks (82), and it is very common among individuals experiencing anxiety, affecting more than 80% of GAD patients (83). In addition, comorbid insomnia is an indicator of increased morbidity and low treatment responsiveness, and it shows a correlation with the severity of worry, fear, and hypervigilance (84). Similarly, there is a two-way relationship between sleep disorders and stress-related disorders including Posttraumatic Stress Disorder (PTSD). This interaction leads to the release of orexin, which most likely contributes to the sustained stress response and increased arousal levels (85). Therefore, orexin regulatory disorders may overlap with insomnia, anxiety, and stress-related diseases, resulting in increased orexin release in the limbic regions of the brain that regulate emotions and stress responses (86). In studies involving patients with anxiety disorders, orexin levels are higher compared to the control group, indicating that the orexin system may be in a hyperactive state (87). Current evidence clearly shows that orexin plays a key role in arousal-type anxiety and suggests that orexin antagonists may be an effective strategy to reduce chronic hyperarousal and interrupt the negative reinforcement cycle of anxiety (83). Therefore, the latest pre-clinical research results confirm and expand the previous application prospects of DORAs as anti-anxiety compounds based on new mechanisms (88).

Similar results have been reported for PTSD and stress-related disorders (89). In a study conducted on healthy volunteers, compared with placebo, 10 mg of suvorexant was associated with a reduced startle reactivity during anticipatory anxiety, but not in the fear or non-threat conditions (90). In two other studies conducted on patients with insomnia, anxiety, and depression, not only improved insomnia symptoms but also anxiety symptoms (91). Treating insomnia symptoms with DORAs may help treat anxiety through the re-balancing of the sleep system.

#### 3.4.3 Insomnia comorbid with schizophrenia

Insomnia symptoms are common in patients with schizophrenia and psychotic spectrum disorders, with the estimated prevalence ranging from 36% to 80% (92). In fact, the relationship between insomnia and schizophrenia may be bidirectional. Insomnia worsens the symptoms of schizophrenia, and vice versa (82).

According to the results of pre-clinical studies, drugs that activate orexin neurons may help treat the negative symptoms and cognitive deficits of schizophrenia (93). DORAs can be used to treat insomnia comorbid with schizophrenia. Sleep regulation can positively impact the treatment of schizophrenia by promoting the restorative effects of sleep on cognitive, metabolic, and immune functions, as well as by reducing excessive arousal (94). Currently, there are no clinical trials on the efficacy of DORAs in treating insomnia comorbid with schizophrenia. However, the use of suvorexant was reported in a case report of a schizophrenia patient treated with aripiprazole (95). In this case, short-term administration effectively treated the insomnia symptoms without impairing cognitive function. Nevertheless, more research is needed on the treatment of insomnia in schizophrenia patients to understand the potential role of DORAs.

#### 3.4.4 Neurodegenerative diseases

Sleep disorders, such as insomnia and sleep-wake rhythm disturbances, are extremely common in patients with Alzheimer’s disease (AD) (96, 97). In fact, changes in sleep may be closely related to the pathophysiological mechanisms of AD. Chronic sleep disruption impairs the clearance function of the glymphatic system, thereby enhancing the accumulation of β-amyloid. It has been reported that the hypothalamic orexin system degenerates in AD patients, and orexin dysfunction may be associated with the pathophysiology of AD through various pathways involving a two-way relationship with amyloid-β. In particular, higher orexin levels in the cerebrospinal fluid have been observed in AD patients, and there are significant correlations between orexin levels and the disruption of sleep macrostructure, cognitive decline, as well as AD biomarkers (i.e., tau protein and amyloid-β). Meanwhile, correlations between orexin A and amyloid-β and phosphorylated tau have also been found in healthy elderly subjects (98).

Based on pre-clinical and observational literature regarding the association between orexin expression dysregulation, β-amyloid accumulation, and tau pathology, a recent study investigated the effects of suvorexant on tau phosphorylation and amyloid-β concentration in a randomized controlled trial and compared the acute administration of two drugs. The trial was conducted in 38 volunteers aged 45–65 years with unimpaired cognitive abilities and without a diagnosis or self-reported sleep disorders. They received either suvorexant doses (10 mg, n = 13; 20 mg, n = 12) or placebo (n = 13) (99). Notably, there were no significant differences in sleep parameters among the three groups. However, compared with the placebo group, in participants who received 20 mg of suvorexant, the ratio of phosphorylated tau-threonine-181 to unphosphorylated tau-threonine-181 (a measure of phosphorylation) decreased by 10-15%, and the amyloid-β levels in the two suvorexant groups were 10-20% lower than those in the placebo group. Reductions were not observed at the other two phosphorylated sites (tau-serine-202 and tau-threonine-217) in the other two studies. Preliminary evidence from case-series studies suggests that suvorexant may be useful in treating nocturnal delirium in AD (100), and ongoing clinical trials aim to evaluate the efficacy of suvorexant in reducing agitation symptoms, aggressive behavior, and sleep disorders in AD patients.

#### 3.4.5 Epilepsy

Epilepsy is associated with an increased risk of various comorbidities, such as sleep disorders (101). It has been reported that insomnia symptoms are highly prevalent among patients with epilepsy (PWE). The prevalence of insomnia diagnosis ranges from 36% to 74% (102). Moreover, it has been shown that the presence of insomnia complaints is closely related to higher levels of depressive symptoms and a poorer quality of life. In addition, sleep deprivation is associated with an increased risk of epileptic seizures in PWE. Therefore, the two-way relationship between epilepsy and sleep has long been recognized and widely described in clinical research, indicating an interdependent relationship between epileptic seizures and sleep disorders, where the occurrence of one condition may exacerbate the other. Specifically, on one hand, the effects of REM sleep on epileptic seizures, interictal epileptiform discharges, and high-frequency oscillations need to be considered. On the other hand, situations such as sleep deficiency, sleep deprivation, sleep instability, and selective REM leading to an increased tendency to epileptic seizures should also be taken into account (103). Thus, it is necessary to study the possible role of neurotransmitter systems involved in sleep regulation, such as the orexin system, in the pathophysiology of epilepsy. In fact, an increasing amount of evidence suggests that the orexinergic system may play a crucial role in epileptic seizures. When orexin levels are high, the tendency to epileptic seizures is higher (i.e., sleep deprivation leading to prolonged wakefulness, or during the transition from REM sleep to wakefulness).

One study has shown that administration of Almorexant (a DORA) can reduce epileptic activity in a mouse model of epilepsy, inhibiting the probability and power of epileptic seizures. However, it also has an off-target effect of reducing motor activity. The selective OX1R antagonist SB-334867 can increase the seizure threshold in mice undergoing acute seizure threshold tests (104).

Meanwhile, the latest research indicates that the pre-seizure natural orexin activity level following systemic administration of the selective OX1R antagonist SB-334867 may be a determinant of seizure intensity, and acute inhibition of natural orexin activity can effectively suppress epileptic seizures (105). However, all evidence regarding the therapeutic potential of DORAs for various comorbid disorders is merely derived from preliminary research findings, and large-sample randomized controlled trials are still required to verify whether such applications can be developed into formal clinical indications.

**Figure 6:**
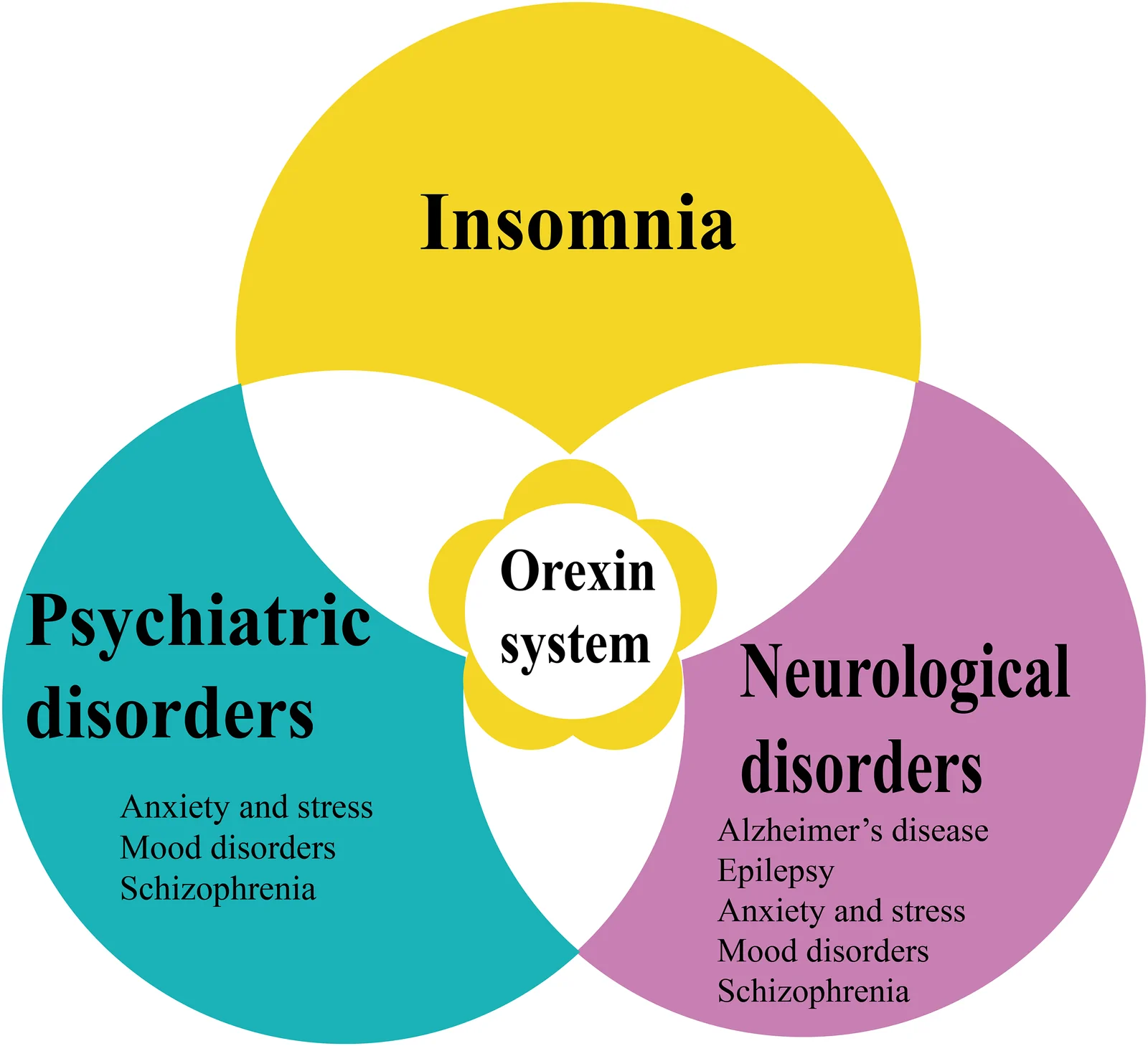
Possible relations linking the orexin system to insomnia as a comorbidity in neurological and psychiatric disorders.

To facilitate the understanding of the relationships among insomnia comorbidities, a brief overview of the relationships among the three hypothesized in sleep medicine is shown in **Figure 6**.

## 4 Conclusion and future perspectives

Intact and ordered sleep architecture serves as a critical foundation for normal brain function and mental health, underscoring the prominent clinical significance of developing novel therapeutics for insomnia. This narrative review systematically integrates existing research evidence on DORAs and delivers a comprehensive discussion covering their pharmacological characteristics, clinical efficacy, and potential therapeutic value across multiple disease contexts. Consistent and robust conclusions have been drawn from multiple Phase III randomized controlled trials: DORAs effectively shorten sleep onset latency and prolong sustained sleep duration in patients with insomnia. Unlike conventional benzodiazepine and non-benzodiazepine GABAergic hypnotics that disrupt slow-wave deep sleep and REM architecture, DORAs act through an inherently distinct mechanism. By selectively blocking the orexin-driven arousal signaling pathway, these agents restore the body’s native physiological sleep rhythm and fully preserve all normal sleep stages. To date, three DORAs — svorexant, lemborexant and daridorexant — have been approved by the U.S. FDA for insomnia treatment, among which daridorexant has also received authorization from the European Medicines Agency (EMA) for patients with chronic insomnia.

From the perspective of individualized clinical decision-making, DORAs exhibit unique advantages over traditional hypnotics for four specific patient populations. First, for individuals on long-term GABAergic sedatives who face high risks of physical and psychological dependence, DORAs carry a low abuse potential and only mild rebound insomnia upon discontinuation. Second, for patients with severely disrupted sleep architecture resulting from chronic benzodiazepine exposure, DORAs maintain intact N3 deep sleep and REM cycles. Third, for patients complicated with mild-to-moderate COPD or OSA, this drug class poses negligible risk of central respiratory depression. Fourth, for insomnia patients suffering persistent daytime impairment including fatigue, cognitive blunting and emotional disturbance, the 50 mg dose of daridorexant ameliorates nocturnal sleep while delivering sustained improvements in daytime alertness and emotional wellbeing.

Although these agents have not yet obtained marketing approval in China, abundant overseas clinical trial data have verified their favorable short- and long-term tolerability. No evident drug tolerance, rebound insomnia or withdrawal reactions were observed among trial participants. Nevertheless, current research has inherent limitations, and long-term real-world follow-up surveillance is required to identify delayed and rare adverse events. Multiple critical evidence gaps still hinder the establishment of standardized clinical medication regimens. No long-term head-to-head trials have directly compared suvorexant, lemborexant and daridorexant, leaving a lack of quantitative data to guide differentiated drug selection in clinical practice. Data regarding long-term rare adverse reactions remain scarce, necessitating real-world cohort studies to uncover hidden safety risks under routine clinical care. Evidence supporting DORAs use in vulnerable groups such as patients with severe psychiatric disorders, severe OSA, advanced COPD, pregnant women and children is insufficient. Furthermore, the safety and pharmacokinetic interactions of DORAs under polypharmacy conditions have not been fully elucidated, making it difficult to provide clear medication guidance for patients with multiple comorbidities receiving combined treatments.

Research directions covered in this paper, including interventions for substance use disorders, adjuvant anti-seizure therapy in epilepsy, modulation of amyloid and tau biomarkers in Alzheimer’s disease, and treatment of primary psychiatric symptoms unaccompanied by insomnia, are merely supported by preclinical mechanistic experiments and small-scale preliminary observations. Without sufficient data from randomized controlled trials, these avenues remain purely research hypotheses rather than established clinical indications. In particular, for insomnia comorbid with schizophrenia, only isolated case reports and neurobiological inferences are available; large-scale clinical trials are absent to confirm definite therapeutic efficacy.

Future clinical and translational research should prioritize filling the above evidence gaps to refine individualized dosing strategies for insomnia patients. Core research priorities include conducting direct comparative trials of the three marketed DORAs, establishing long-term real-world platforms for drug safety monitoring, exploring optimal dosages for vulnerable populations, and formulating standardized management protocols for drug-drug interactions. Rigorous clinical trials are also warranted to validate whether the aforementioned hypothetical therapeutic targets can be translated into formal clinical interventions. In summary, DORAs represent a transformative paradigm in insomnia pharmacotherapy. As supporting clinical evidence continues to accumulate, this class of agents will enable more comprehensive, individualized care for diverse insomnia populations and facilitate the research, development and clinical translation of next-generation sleep disorder therapeutics.
